# Assessment of reporting quality of conference abstracts in sports injury prevention according to CONSORT and STROBE criteria and their subsequent publication rate as full papers

**DOI:** 10.1186/1471-2288-12-47

**Published:** 2012-04-11

**Authors:** Uzung Yoon, Karsten Knobloch

**Affiliations:** 1 Surgery Department, New York Hospital Queens, Flushing, NY, USA; 2 Plastic, Hand and Reconstructive Surgery, Hannover Medical School, Carl-Neuberg-Str. 1, 30625 Hannover, Germany

**Keywords:** Conference, Abstract, Quality, Study, Peer-review

## Abstract

**Background:**

The preliminary results of a study are usually presented as an abstract in conference meetings. The reporting quality of those abstracts and the relationship between their study designs and full paper publication rate is unknown. We hypothesized that randomized controlled trials are more likely to be published as full papers than observational studies.

**Methods:**

154 oral abstracts presented at the World Congress of Sports Injury Prevention 2005 Oslo and the corresponding full paper publication were identified and analysed. The main outcome measures were frequency of publication, time to publication, impact factor, CONSORT (for Consolidated Standards of Reporting Trials) score, STROBE (for Strengthening the Reporting of Observational Studies in Epidemiology) score, and minor and major inconsistencies between the abstract and the full paper publication.

**Results:**

Overall, 76 of the 154 (49%) presented abstracts were published as full papers in a peer-reviewed journal with an impact factor of 1.946 ± 0.812. No significant difference existed between the impact factor for randomized controlled trials (2.122 ± 1.015) and observational studies (1.913 ± 0.765, p = 0.469). The full papers for the randomized controlled trials were published after an average (SD) of 17 months (± 13 months); for observational studies, the average (SD) was 12 months (± 14 months) (p = 0.323). A trend was observed in this study that a higher percentage of randomized controlled trial abstracts were published as full papers (71% vs. 47%, p = 0.078) than observational trials. The reporting quality of abstracts, published as full papers, significantly increased compared to conference abstracts both in randomized control studies (CONSORT: 5.7 ± 0.7 to 7.2 ± 1.3; p = 0.018, CI -2.7 to -0.32) and in observational studies (STROBE: 8.2 ± 1.3 to 8.6 ± 1.4; p = 0.007, CI -0.63 to -0.10). All of the published abstracts had at least one minor inconsistency (title, authors, research center, outcome presentation, conclusion), while 65% had at least major inconsistencies (study objective, hypothesis, study design, primary outcome measures, sample size, statistical analysis, results, SD/CI). Comparing the results of conference and full paper; results changed in 90% vs. 68% (randomized, controlled studies versus observational studies); data were added (full paper reported more result data) in 60% vs. 30%, and deleted (full paper reported fewer result data) in 40% vs. 30%.

**Conclusions:**

No significant differences with respect to type of study (randomized controlled versus observational), impact factor, and time to publication existed for the likelihood that a World Congress of Sports Injury conference abstract could be published as a full paper.

## Background

A considerable proportion of health care research are first presented at conferences and meetings, and published as abstracts in the proceedings. Although many of the conference abstracts are subsequently published as full papers in peer-reviewed journals, the data presented in the abstracts at these conferences may be inconsistent with the manuscripts of the final published papers. Conference abstracts may present preliminary results of an ongoing study which might be the reason for data inconsistency compared to the corresponding full paper publication.

Publication rates of 32% to 67% have been published for orthopedic meetings in the past [1]. However, to date there are no reports analyzing the reporting quality of conference abstracts and its corresponding published full paper abstracts. In addition, there are no analysis assessing the correlation between the study type (randomized, controlled trial, observational study, and so forth) and the publication rate.

The 1st World Congress on Sports Injury Prevention was held at the Holmenkollen Park Hotel in Oslo, Norway in June 2005. All abstracts were published as supplements of the *British Journal of Sports Medicine*. We hypothesized that abstracts for randomized controlled trials (which are clinical trials that are based on a higher level of evidence than observational studies) were more likely to be published than were observational studies following the abstract presentation at the World Conference of Sports Injury Prevention 2005 in Oslo.

We evaluated the publication rate and data consistency between the conference abstracts and the corresponding full paper publication presented at the first *World Congress of Sports Injury Prevention *in Oslo 2005. We used the CONSORT (Consolidated Standards for Reporting Trials) criteria for randomized-controlled trials [2] and the STROBE (Strengthening the Reporting of Observational Studies in Epidemiology) criteria for observational studies [3] and for minor and major inconsistencies. To date, there are no publications reporting the quantity of each study design in conference abstract presentations and the relationship between the study design and whether the studies were ultimately published as full papers.

## Methods

All 154 oral abstracts from the *1st World Conference on Sports Injury Prevention in Oslo 2005 *were analyzed in a database. PubMed and Medline online searches were performed as continuous follow up over a 42-month period to ascertain whether the presented oral abstracts were followed by full paper publication. These searches were done using the authors names (first, second, and last authors, each searched individually); then they were compared with probable keywords and title phrases of the abstract. When full paper publications were identified, the title of the full paper publication, the name(s) of authors, the location(s) where research was conducted, study design, sample size, follow-up duration, number of withdrawals, and study results data were compared with the information available from the conference abstracts. Verification of conference abstracts and their corresponding full paper was performed. The following items were verified: same title and author name(s); same location(s) for research; consistent study designs, follow-up durations, and numbers of withdrawals; and no major differences in study results data. Multiple full paper publications for a single abstract were also identified; just the first full paper publication was used for analysis.

For each conference abstract and its corresponding full paper publication, CONSORT abstract criteria for all randomized (Additional file 1: Appendix 1), controlled trials and STROBE for all observational studies (Additional file 1: Appendix 2) were applied. Scores were calculated for 17 CONSORT criteria and 22 STROBE criteria for all conference abstracts; two investigators independently assessed each conference abstract and the corresponding full paper publication abstract and calculated the score. The mean score of both investigators in each abstract was taken. Inter-rater variability was 6%.

Clinical studies with known study designs were separated from experimental studies. A randomized controlled trial was identified if the abstract described a prospective study in which individuals were allocated at random to an intervention or a control group. Observational studies included cohort, case-control, and cross-sectional studies; systematic reviews; and case series and case reports. If the study design was still unclear after attempts to match it with the formats outlined above, abstracts were analyzed by both researchers together (UY and KK). Among the facets of each study that were re-analyzed were how the study was performed; patient recruitment; study duration and setting; intervention and follow up; statistical data, analysis and data in the results. Furthermore, the entire abstract was searched for specific words like "prevalence", "placebo", "blinded", "random", "questionnaire", "lab techniques (PCR, Western/Southern/Northern-blot)", "odds ratio", and "relative risk" to determine the study design.

In addition to the above, the names and numbers of authors; the numbers of centers (location where research was conducted); the objective and/or hypothesis; the author's interpretation of data; sample size and study results; standard deviations (SD) and confidence intervals (CI) (actual numbers); location(s) of study; and method of study (randomized controlled trial, observational trial, statistical analysis, primary outcomes) were all recorded. For randomized controlled trials, follow-up duration, method of randomization, blinding, use intent-to-treat analysis, and number of withdrawals/dropouts were also noted.

### Outcome measures

The scores of 17 CONSORT criteria and 22 STROBE criteria for reporting were determined. Publication rate, publication time, minor and major inconsistencies between conference abstracts, and whether the study was ultimately published as a full paper were measured. Minor inconsistencies included differences in title, authors, research center, presentation of all outcomes (p-value, confidence interval, Pearson), and authors' interpretation of data (conclusion). Major inconsistencies included discrepancies in study objective and/or hypothesis, study design, primary outcome measures, sample size, statistical analysis, results, and standard deviations/confidence intervals. The primary outcome was defined as the main outcome reported in an abstract. If the amount of reported result data in the abstract did not match the full paper it was described as "results different". Mismatches in the amount of study result data presented between the abstract and the full paper were reported in four categories. If the full paper reported fewer result data than were presented in the abstract, it was reported as "data deleted". A full paper with more result data than were present in the abstract was reported as "data added"; if completely result different data were reported in the full paper in comparison to the abstract, it was described as "results completely changed". For the specific instance in which just the standard deviation, confidence interval, injury rate, or incidence changed, it was described as "SD/CI, Injury rate, Incidence changed". The impact factor, which is dependent on the year and the journal's distribution of the published abstracts, was also determined.

### Statistics

Descriptive statistics consisted of the calculation of frequencies and percentages. Clopper and Pearson analysis were used for binomial proportion confidence interval. The T-test was used to compare minor and major inconsistencies, the CONSORT and STROBE score, and the impact factors between randomized, controlled trials and observational studies. Chi-squared tests were used to compare publication rates, odds ratios, 95% confidence intervals, and p values according to the different countries. Statistical significance was defined as p < 0.05. Data were analyzed using the SPSS statistical software package Version 14.0 and StatXact version 6.

## Results

### Publication rate and impact factor

154 conference abstracts were analyzed. 14 (9%) were randomized controlled trials; 135 (88%) were observational studies; and 5 (3%) were experimental studies. Overall, 76 (49%) of the 154 conference abstracts were published as full papers in peer-reviewed journals, with an impact factor of 1.946 ± 0.812. 71% (10) of RCTs, 47% (63) of observational studies, and 60% (3) of experimental studies were full paper published. There was no significant difference between the impact factor for randomized clinical trials (2.122 ± 1.015) compared to that for observational studies (1.913 ± 0.765, p = 0.469). For randomized controlled trials the full paper was published on an average (SD) of 17 months (± 13 months), compared to 12 months (± 14 months) for observational studies (p = 0.323). There was a trend towards more percentage of randomized controlled trial abstracts being published as full papers (71% vs. 47%, p = 0.078), but there was no statistical significance. Two abstracts were associated with multiple full paper publications.

**Figure 1:**
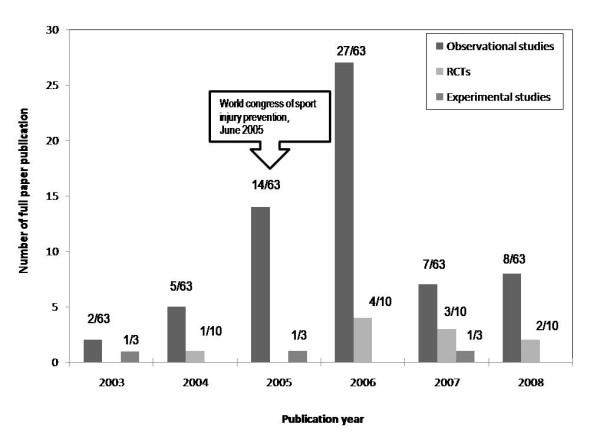
**Years in which full papers were published after presented as abstracts at the World Congress of Sports Injury Prevention in 2005 in Oslo**. (Overall full paper publication: 76; observational study: n = 63, RCT: n = 10, experimental study: 3).

Twelve conference abstracts were published as full papers prior to the presentation at the Congress in June 2005 (-11 months ± 10 months). Most conference abstracts were published as full papers during the first three years after the meeting; 40.8% after the first year, 15.8% in the second year; and 13.2% in the third year (Figure 1). About one-third of the conference abstracts were published as full papers in the *American Journal of Sports Medicine *or the *British Journal of Sport Medicine*, with 16% for each (CI 8 to 26), respectively, followed by the *Scandinavian Journal of Medicine & Science in Sports *(10%, CI 4 to 18, Table 1).

**Table 1:** Published rate per journal in percentage

Ranking	Name of published Journal 2003-2008	Impact factor 2007	Overall (N, %)	95% confidence interval	RCT (N, %)	Observational Study (N, %)
1	*American Journal of Sports Medicine*	3,397	12 (15.8%)	8-26	3(25%)	9 (75%)

2	*British Journal of Sport Medicine*	2,463	12 (15.8%)	8-26	0 (0%)	12 (100%)

3	*Scandinavian Journal of Medicine & Science in Sports*	2,295	7 (9.2%)	4-18	1(14%)	6 (86%)

4	*The Journal of Science and Medicine in Sport*	1,091	4 (5.3%)	1-13	2 (50%)	2 (50%)

4	*Knee Surgery, Sports Traumatology, Arthroscopy*	1,626	4 (5.3%)	1-13	1(25%)	3 (75%)

4	*Clinical Journal of Sport Medicine*	1,663	4 (5.3%)	1-13	1(25%)	3 (75%)

7	*International Journal of Sports Medicine*	1,524	3 (3.9%)	0.8-11	0 (0%)	3 (100%)

8	*The Journal of Strength and Conditioning Research*	0,453	2 (2.6%)	0.3-9.2	0 (0%)	2 (100%)

8	*Sportverletzung · Sportschaden*	0,170	2 (2.6%)	0.3-9.2	0 (0%)	2 (100%)

8	*Journal of Orthopaedic Research*	2,437	2 (2.6%)	0.3-9.2	0 (0%)	2 (100%)

8	*Journal of Biomechanics*	2,897	2 (2.6%)	0.3-9.2	0 (0%)	2 (100%)

8	*Journal of Applied Physiology*	3,632	2 (2.6%)	0.3-9.2	0 (0%)	2 (100%)

### Publication rates according to country

Abstracts originated from 25 countries: 53% were from Europe (n = 81), 24% from Oceania (n = 37), 17% from North America (n = 26), 5% from Asia (n = 8) and 1.3% from Africa (n = 2). All randomized, controlled trial conference abstracts from Norway, the United States, Canada, and Sweden were published as full papers (Table 2).

**Table 2:** Publication rates according to the country

Country	RCT (N)	No. of abstracts expanded into full-text publication	Observational studies (N)	No. of abstracts expanded into full-text publication	Odds ratio	p
Australia	4	2 (50%)	25	15 (60%)	1.94 (0.8-4.69)	0.139

New Zealand	0	0 (0%)	7	0 (0%)	-	0.011

USA	1	1 (100%)	13	9 (69%)	2.83 (0.83-9.7)	0.086

Canada	1	1 (100%)	10	7 (70%)	2.88 (0.71-11.6)	0.124

Germany	0	0 (0%)	12	4 (33%)	0.54 (0.16-1.90)	0.332

Norway	3	3 (100%)	11	4 (36%)	0.63 (0.18-2.26)	0.475

Switzerland	0	0 (0%)	7	3 (43%)	0.85 (0.18-4)	0.836

Sweden	1	1 (100%)	6	4 (67%)	2.37 (0.42-13.42)	0.315

Spain	0	0 (0%)	5	0 (0%)	-	0.033

Japan	0	0 (0%)	5	2 (40%)	0.75 (0.12-4.66)	0.761

Denmark	2	1 (50%)	5	4 (80%)	4.81 (0.52-44.3)	0.128

Other	2	1 (50%)	34	14 (44%)	0.83 (0.38-1.77)	0.626

Total	14	10	140	66		

### Changes in quality from conference abstract to full text publication

The quality in reporting increased from the conference abstract to the full paper publication abstract in both randomized controlled trials and observational studies. The CONSORT score for randomized controlled trials increased from 5.7 ± 0.7 to 7.2 ± 1.3 (p = 0.018, CI -2.7 to -0.32, score range 1-17). Improvements were reported in trial designs, participants, and intervention. The STROBE score for observational studies increased from 8.2 ± 1.3 to 8.6 ± 1.4 (p = 0.007, CI -0.63 to -0.10 score range 1-22). Improvements were seen in titles, background, and objectives (Tables 3 and 4).

**Table 3:** Quality of reporting of conference abstracts and full paper publication abstract according to the CONSORT criteria for randomized-control trials

	Conference abstract N = 14		Full paper publication abstract N = 10	
	%	N	%	N

**Title-randomised**	21	3	50	5

**Authors**	0	0	0	0

**Trial design**	57	8	90	9

**Participants**	57	8	50	5

**Interventions**	86	12	80	8

**Objective/Hypothesis**	79	11	100	10

**Main Outcome**	0	0	20	2

**Randomisation**	14	2	10	1

**Blinding**	0	0	0	0

**Number Randomised**	64	9	100	10

**Recruitment**	0	0	0	0

**Numbers analysed**	14	2	20	2

**Outcome**	86	12	100	10

**Harms**	0	0	0	0

**Conclusion**	100	14	100	10

**Trial registration**	0	0	0	0

**Funding**	0	0	0	0

**Table 4:** Quality of reporting of conference abstracts and full paper publication abstract according to the STROBE criteria for observational trials

	Conference abstract N = 135		Full paper publication abstract N = 63	
	**%**	**N**	**%**	**N**

**Title and abstract**	10	14	11	7

**Background/rationale**	53	72	46	29

**Objectives**	82	111	95	60

**Study design**	25	34	46	29

**Setting**	44	59	49	31

**Participants**	84	113	87	55

**Variables**	21	28	35	22

**Data sources/measurement**	78	105	81	51

**Bias**	0	0	0	0

**Study size**	0	0	0	0

**Quantitative variables**	0	0	0	0

**Statistical methods**	9	12	8	5

**Participants**	1	1	6	4

**Descriptive data**	0	0	0	0

**Outcome data**	96	129	98	62

**Main result**	95	128	97	61

**Other analysis**	0	0	0	0

**Key result**	96	130	98	62

**Limitation**	0	0	0	0

**Interpretation**	96	129	98	62

**Generalizability**	0	0	0	0

**Funding**	0	0	0	0

### Minor and major inconsistencies

No significant differences in major and minor inconsistencies according to randomized controlled trials (2.6 ± 0.7 vs. 2.8 ± 1, p = 0.488) and observational studies (1.9 ± 1.3 vs. 1.6 ± 1.4, p = 0.656) were reported. All of the published abstracts had at least one minor inconsistency (RCT: 10 (100%), observational study: 63 (100%)); 65% of the abstracts had at least one major inconsistency (RCT: 8 (80%), observational study: 41 (54%)). Minor inconsistencies were much more prevalent and included changes in the presentation of the outcomes (100% vs. 95%), changes in title (80% vs. 87%), changes in authorship (50% vs. 57%), changes in interpretation (20% vs. 25%), and changes in research center (10% vs. 17%, Table 5). The most common major inconsistencies included changes in results and sample sizes. For the changes in results (randomized controlled trials vs. observational studies, respectively), data were added (60% vs. 30%), deleted (40% vs. 30%) and completely changed (0% vs. 5%) in abstracts compared to the full paper publications (Table 6). Table 7 shows the number of inconsistencies per conference abstract/full paper pairing. The respective differences for study objective/hypothesis, study design, and primary outcome measures were (0% vs. 11%), (0% vs. 13%) and (10% vs. 16%) (for randomized, controlled trials vs. observational studies).

**Table 5:** Minor inconsistencies between conference abstract and final full text publication (n = 76) for all studies, as well as for randomized-controlled trials (RCT) and observational studies

	RCT		Observational studies		Experimental study design	Total Publications		
	**Percentage %**	**95% CI**	**Percentage %**	**95% CI**	**N**	**Percentage %**	**95% CI**	**n**

**Minor inconsistency**	10 (100%)	69-100	63 (100%)	94-100	3	76 (100%)	77-94	76

Title was different	8 (80%)	44-97	55 (87%)	77-94	0	66 (87%)	6-23	10

Author was different	5 (50%)	19-81	36 (57%)	44-70	2	42 (55%)	43-67	34

Research center was different	1 (10%)	0.2-45	11 (17%)	9-29	3	36 (16%)	36-60	64

Presentation was different	10 (100%)	69-100	60 (95%)	87-99	0	73 (96%)	89-99	3

Interpretation was different	2 (20%)	3-56	16 (25%)	15-38	2	19 (25%)	16-36	57

**Table 6:** Major inconsistencies between conference abstract and final publication (n = 76) for all studies, as well as for randomized-controlled trials (RCT) and observational studies

	RCT		Observational studies		Experimental study design	Total Publications		
	**Percentage %**	**95% CI**	**Percentage %**	**95% CI**	**N**		**95% CI**	**n**

**Major inconsistency**	8 (80%)	44-97	41 (54%)	52-77	0	49 (65%)	53-75	49

Study hypothesis different	0 (0%)	0-30	7 (11%)	5-22	3	10 (13%)	6-23	69

Study design different	0 (0%)	0-30	8 (13%)	6-24	3	11 (15%)	7-24	68

Primary outcome measure different	1 (10%)	0-46	10 (16%)	8-27	3	14(18%)	10-29	65

Sample size different	7 (70%)	35-93	25 (40%)	28-53	0	32 (42%)	31-54	32

*Increased*	4 (40%)	12-74	15 (24%)	14-36	0	19 (25%)	23-45	19

*Decreased*	3 (30%)	7-65	12 (19%)	10-31	0	15 (20%)	11-30	15

Not stated*	0 (%)	0-30	4 (6%)	2-16	2	6 (8%)	3-16	6

Sample size same	3 (30%)	7-65	32 (51%)	38-64	1	36 (48%)	36-59	36

Statistic Not stated	9 (90%)	56-100	59 (94%)	85-98	3	71 (93%)	85-98	71

*Same*	1 (10%)	0-46	2 (3%)	0-11	0	3 (4%)	1-11	3

Result different	9 (90%)	56-100	43 (68%)	55-79	3	55 (72%)	60-82	24

*Data added*	6 (60%)	26-88	19 (30%)	19-43	0	25 (33%)	23-45	25

*Data deleted*	4 (40%)	12-74	19 (30%)	19-43	0	23 (30%)	20-42	23

*Complete changed*	0	0-30	3 (5%)	1-13	0	3 (4%)	1-11	3

*SD/CI/, Injury rate, Incidence* *changed*	3 (30%)	7-65	15 (24%)	14-36	0	18 (24%)	15-35	18

*in both conference and full-text abstract

**Table 7:** Number of inconsistencies by study design.

Number of minor inconsistencies per abstract/full paper pair	RCT	Observational	*Overall*
0	0	0	*0*

1	0	7	*7*

2	5	18	*23*

3	4	23	*27*

4	1	15	*16*

5	0	0	*0*

Total	10	63	

**Number of major inconsistencies per abstract/full paper pair**	**RCT**	**Observational**	***Overall***

0	0	13	*13*

1	3	7	*10*

2	2	9	*11*

3	2	9	*16*

4	1	1	*1*

5	0	2	*2*

*Total*	*8*	*41*	

## Discussion

### The principal findings of this study are as follows

Only about half of the abstracts presented at the 1^st ^World Conference of Sports Injury Prevention were published as full papers in a peer-reviewed journal within three years of their conference presentation. Although we encountered a trend towards more percentage of RCT abstracts being published as full papers rather than observational trial abstracts, this difference did not reach statistical significance. The impact factor of full paper published randomized controlled trials was 2.122, similar to that of observational studies with 1.913, subsequent to the Oslo conference presentation. Time to full paper publication did not differ significantly between randomized, controlled trials (17 months ± 13 months) and observational studies (12 months ± 14 months). Notably, 12 abstracts were published as full papers prior to the Oslo conference. Thus, the primary hypothesis has to be rejected.

The above observations should be discussed in detail. We thought that the study design of a given conference abstract presented at an international sports injury prevention conference influenced the likelihood of publication of a subsequent full paper. Given the enormous complexity, costs, and effort spent performing a randomized, controlled trial in sports injury prevention rather than an observational study, we believed that the rate of publication of abstracts presented as randomized clinical trials should be higher than for observational studies. However, there was no statistical significance of the full paper publication rate between RCTs and observational studies. It is possible that this is due to the small amount of RCTs. Time to full paper publication and impact factor also did not differ significantly.

The wide variation in full paper publication rates for abstracts presented at diverse medical and scientific congresses has been studied and extensively reported. For example, Bhandari et al [4] noted a publication rate of 34% for orthopedic conference abstracts and inconsistencies in the primary outcome measure in 14% and 19% of results between the first presentation of the abstracts in 1996 and final full paper publication (4.7-year follow-up). Kleweno [5] analyzed the American Orthopaedic Society for Sports Medicine (AOSSM) abstracts and subsequent full papers from 1999 to 2001 regarding potential minor and major inconsistencies. While 59.4% of the AOSSM abstracts were published as full papers within 21 months after presentation, minor and major inconsistencies were evident in more than half of the full papers compared with the initial abstract presented. Comparing the distribution of different study designs in sports injury prevention conferences, randomized, controlled trials represent about 10% of all oral presentations [6]. Our findings showed a 49.4% full paper publication rate within three years and major inconsistencies in 65% of abstract/full paper pairings are comparable with these studies. However, these studies did not analyze the impact of the study design in the initial abstract on the likelihood of subsequent publication.

Sprague [7] has highlighted three main potential reasons for why conference abstracts are not published as full papers:

1. not enough time to prepare a manuscript for full paper publication,

2. the studies are ongoing, and

3. relationships with co-authors could cause a barrier to final publication.

Another explanation, for an abstract that did not lead to a peer-reviewed journal full paper publication, is that the project did not survive the peer-review process of a journal, even if it has passed the peer review process for the conference. While abstracts submitted for scientific meetings are typically graded by a review committee, the details on the research methodology contained within the short abstract are at best very limited. In some circumstances, subjecting a full manuscript to peer review might reveal significant methodological flaws, preventing the abstract from appearing as a full paper [8].

Clarity of reporting is a prerequisite to evaluation. Clear, transparent, and accurate reporting in abstracts is important as well. To increase the rate of full paper publication and to decrease both major and minor inconsistencies in conference meeting abstracts, a substantial and comprehensive use of the CONSORT criteria for randomized controlled trials and the STROBE criteria for observational studies should be endorsed [2,3,9].

We strongly believe that STROBE and CONSORT recommendations on reporting of research might substantially increase the quality in reporting sports injury conference abstracts, potentially leading to a higher rate of full paper publications in the future. However, the statements should not be interpreted as an attempt to prescribe the reporting of observational research in a rigid format. The checklist items should be addressed in sufficient detail and with clarity at some point in an article, but the order and format for presenting information depends on author preferences, journal style, and the traditions of the research field [3].

### Limitations

Several study limitations should be noted. First, we evaluated the quality of reporting, which is not the same as the methodologic quality of the study. It is possible that a poorly reported study is well designed and executed, and a well-reported one may have several shortcomings. Second, we were only able to evaluate the information presented in the conference abstract. It is possible that due to the limited information in abstracts, study designs could have been misinterpreted, or there could have been insufficient comparison of results data. In particular, the reported primary outcome in an abstract may not be the primary outcome of the study design. In some instances, a full paper publication may report an outcome different from the primary outcome. For example, conference abstracts are more likely to report interim analyses than are full paper publications [9]. And also a single study could be published as more than one abstracts in which they could conclude different results. Therefore (which served as a constraint in our study), we defined the primary outcome as the main outcome reported in an abstract. Given the suggestions of the conference committee regarding the abstract format and the restricted word count, one must note that the implementation of more comprehensive reporting in abstracts might be limited by the organizers' requirements of abstract format.

Another separate possible limitation in our analysis was that we used only the first full paper publication for analysis in instances where abstracts were associated with multiple publications. However, the amount of multiple full paper publication is small and would not significantly change our results. It is also possible that multiple abstracts existed for one full paper publication and that these abstracts were from presentations given at other meetings.

The median follow-up time was 42 months, in line with the fact that most abstracts followed by a full paper article were published within 36 to 48 months [10,11].

It is possible that some abstracts were full-paper published after our literature search, or that some have yet to be published, which would lead to underestimations in our final full paper publication rate. We may also have missed full paper publications that are not indexed in the PubMed database.

Finally, the CONSORT criteria was suggested primarily for randomized controlled trials, which in this particular case only accounted for about 10% of the study designs in conference abstracts. We also acknowledge that STROBE is currently limited to three main observational study designs: cohort, case-control, and cross-sectional studies. No statements or checklists for experimental or other study designs were available.

## Conclusions

After presentation at the World Congress of Sports Injury Prevention in 2005, only about half of the abstracts were published as full papers in a peer-reviewed journal within three years of the conference presentation. No significant difference was observed in the likelihood of full paper publication for randomized clinical trials versus observational trials.

## Competing interests

The authors declare that they have no competing interests. No author received internal or external funding.

## Authors' contributions

KK developed the idea, analyzed all abstracts and wrote the manuscript. UY co-analyzed the data and co-wrote the manuscript. All authors read and approved the final manuscript.

## Pre-publication history

The pre-publication history for this paper can be accessed here:

http://www.biomedcentral.com/1471-2288/12/47/prepub

## Supplementary Material

Additional file 1 **Appendix 1**. CONSORT abstract checklist for reporting in journal and conference abstracts. **Appendix 2**. STROBE-Checklist for observational studies. Click here for file

## References

[B1] Scherer RW Langenberg P von Elm E Full publication of results initially presented in abstracts Cochrane Database Syst Rev 2007 18 MR000005 17443628 10.1002/14651858.MR000005.pub3

[B2] Hopewell S Clarke M Moher D Wager E Middleton P Altman DG Schulz KF CONSORT group CONSORT for reporting randomised trials in journal and conference abstracts Lancet 2008 371 281 283 10.1016/S0140-6736(07)61835-2 18221781

[B3] von Elm E Altman DG Egger M Pocock SJ Gøtzsche PC Vandenbroucke JP STROBE Initiative The Strengthening the Reporting of Observational Studies in Epidemiology (STROBE) statement: guidelines for reporting observational studies J Clin Epidemiol 2008 61 344 10.1016/j.jclinepi.2007.11.008 18313558

[B4] Bhandari M Devereaux PJ Guyatt GH Cook DJ Swiontkowski MF Sprague S Schemitsch EH An observational study of orthopaedic abstracts and subsequent full-text publications J Bone Joint Surg Am 2002 84 615 621 11940624 10.2106/00004623-200204000-00017

[B5] Kleweno CP Bryant WK Jacir AM Levine WN Ahmad CS Discrepancies and Rates of Publication in Orthopaedic Sports Medicine Abstracts Am J Sports Med 2008 36 1875 1879 10.1177/0363546508319054 18667625

[B6] Yoon U Knobloch K Quality of reporting in sports injury prevention abstracts according to the CONSORT and STROBE criteria-an analysis of the World Congress of Sports Injury Prevention in 2005 and 2008 Br J Sports Med 2009 in press 10.1136/bjsm.2008.053876 19656768

[B7] Sprague S Bhandari M Devereaux PJ Swiontkowski MF Tornetta P Cook DJ Dirschl D Schemitsch EH Guyatt GH Barriers to full-text publication following presentation of abstracts at annual orthopaedic meetings J Bone Joint Surg Am 2003 85-A 158 163 12533587 10.2106/00004623-200301000-00024

[B8] Eck JC Publication rates of abstracts presented at Biennial Meetings of the International Society of Arthroscopy, Knee Surgery and Orthopaedic Sports Medicine Knee Surg Sports Traumatol Arthrosc 2005 13 426 429 10.1007/s00167-004-0559-8 16010585

[B9] Hopewell S Clarke M Askie L Reporting of trials presented in conference abstracts needs to be improved J Clin Epidemiol 2006 59 681 684 10.1016/j.jclinepi.2005.09.016 16765270

[B10] Hamlet WP Fletcher A Meals RA Publication patterns of papers presented at the annual meeting of the American Academy of Orthopaedic Surgeons J Bone Joint Surg Am 1997 79 1138 1143 9278072 10.2106/00004623-199708000-00004

[B11] Wang JC Yoo S Delamarter RB The publication rates of presentations at major Spine Specialty Society meetings (NASS, SRS, ISSLS) Spine 1999 24 425 427 10.1097/00007632-199903010-00002 10084177

